# Mitogenome sequence of a Black Sea isolate of the kinetoplastid *Bodo saltans*

**DOI:** 10.1080/23802359.2018.1507654

**Published:** 2018-09-10

**Authors:** Romain Gastineau, Claude Lemieux, Monique Turmel, Nikolaï A. Davidovich, Olga I. Davidovich, Jean-Luc Mouget, Andrzej Witkowski

**Affiliations:** a Natural Sciences Research and Educational Center and Palaeoceanology Unit, Faculty of Geosciences, University of Szczecin, Szczecin, Poland;; b Département de biochimie, de microbiologie et de bio-informatique, Institut de Biologie Intégrative et des Systèmes, Université Laval, Québec, Canada;; c T. I. Vyasemsky Karadag scientific station – Nature Reserve, Feodosiya, Russia;; d Mer, Molécules, Santé (MMS), FR CNRS 3473 IUML, Le Mans Université, Le Mans, France

**Keywords:** *Bodo saltans*, kinetoplastida, maxicircle, next generation seqencing, Black Sea

## Abstract

We obtained the mitogenome sequence of a Black Sea isolate of the kinetoplastid *Bodo saltans*. This sequence consists of two contigs totaling 24,925 bp and encodes ten protein-coding genes, one conserved ORF and one rRNA gene. Alignment of the Black Sea mitogenome with the limited sequence data currently available in public databases for another strain of *B. saltans* revealed significant genetic divergence between the two isolates. Maximum likelihood phylogenetic inference clearly resolved the Bodonidae from the Trypanosomatidae.

Kinetoplastida (phylum Euglenozoa) are protists whose distinctive feature is the presence of a kinetoplast, a network made of several circular DNA strands divided into minicircles and maxicircles. The maxicircles code for mitochondrial proteins, while the minicircles play a role in the maturation of the mRNA coded by the maxicircles (Lukeš et al. 2002). Kinetoplastids are commonly divided into parasitic and free-living (Opperdoes et al., 2016), the parasitic species being responsible of serious diseases like trypanosomiasis and leishmaniasis. *Bodo saltans* is a free-living species often found in polluted area, sewage water and eutrophic environment, where it feeds on bacteria (Mitchell et al. 1988; Doležel et al. 2000; Jackson et al. 2008). Only a 4040 bp fragment of the mitogenome from a freshwater *B. saltans* strain (Lake Constance) is currently available in GenBank (AF041263, Blom et al. 1998), and a genome project failed to reveal the putatively missing mitochondrial sequences (Jackson et al. 2008; ftp://ftp.sanger.ac.uk/pub/project/pathogens/Bodo/saltans/).

We sequenced total DNA from a *B. saltans* strain we isolated from Kazachia Bay in the Black Sea (44°34′19″N, 33°24′07″E). This strain was cultured in F/2 medium (20% salinity) enriched with yeast extract. Cells were ground in liquid nitrogen and DNA was extracted essentially as reported by Doyle and Doyle (1990). An Illumina library of 300 bp DNA inserts was prepared and sequenced on the HiSeq 4000 platform by the Beijing Genomic Institute. A total of 32 million paired-end reads of 150 bp were obtained and assembled using SPAdes 3.12.0 (Bankevich et al. 2012) and a k-mer of 127.

Analysis of the 18S rRNA gene sequence retrieved from the assembled contigs (MH614643) revealed that it is identical to that of the *B. saltans* strain isolated from Gelendzhik, a site also located on the Northern part of the Black Sea (DQ207571, Schekenbach et al. 2006). Mitogenome sequences were recovered as two contigs totaling 24,925 bp in size. The 17,936 bp contig (MH614645) contains ten protein-coding genes (*ND8, ND2, ND1*, *cox*1 *cox*2, *ND5, ND4, ND7*, *cox*3, *ATP6*, and the conserved ORF MURF2) as well as a 540 bp fragment of the 12S rRNA gene (see De La Cruz et al. 1985; Horváth et al. 1990), while the other contig of 6989 bp (MH614644) contains only *cob*. The 9S rRNA and *rps12* genes were not detected, but the latter could be encoded in a pan-edited G-rich region as reported for the *Leishmania tarentolae* kinetoplast maxicircle DNA (Maslov et al. 1992). The portion of the 17,936 bp contig spanning *cox1* is co-linear with the previously reported sequence from the Lake Constance strain (AF041263). This gene shows 80.5% sequence identity between the two strains, with the Black Sea coding sequence starting with a TTG codon instead of ATG.

The total length of our mitogenome assembly falls in the same size range as those reported for complete kinetoplast maxicircles (e.g. DQ343646 in Westenberger et al. 2006). However, Blom et al (1998) estimated that the *B. saltans* maxicircle could be as large as 70 kb, and recent long-read sequencing suggests that some kinetoplast maxicircles (CP022652, CM008275) could be up to 50 kb in size. It is likely that the sequences spanning the gaps between the two contigs we recovered contain abundant repeats, which prevented us from assembling the complete maxicircle using short reads. Future studies of the *B. saltans* mitogenome may thus benefit from third generation sequencing.

The sequences of a segment of about 3450 bp encoding the *cox*1 and *cox*2 genes of several kinetoplast maxicircles were aligned and a maximum likelihood tree was inferred using MEGA6 (Tamura et al. 2013). As expected, the two *B. saltans* strains were recovered in a highly supported clade that is distinct from the cluster containing the Trypanosomatidae (Figure 1).

**Figure 1.**
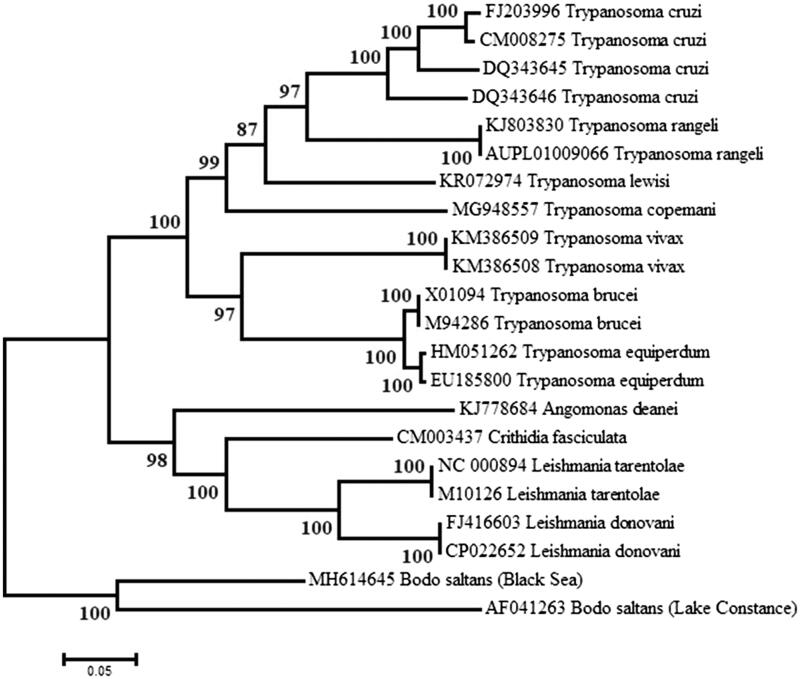
Maximum Likelihood tree of the *cox1-cox2* segment from several kinetoplasts’ maxicircles. The tree with the highest likelihood is shown. Numbers next to nodes are support values obtained after 1000 bootstrap replicates.
